# Diabetes Care Provision in UK Primary Care Practices

**DOI:** 10.1371/journal.pone.0041562

**Published:** 2012-07-30

**Authors:** Gillian Hawthorne, Susan Hrisos, Elaine Stamp, Marko Elovainio, Jill J. Francis, Jeremy M. Grimshaw, Margaret Hunter, Marie Johnston, Justin Presseau, Nick Steen, Martin P. Eccles

**Affiliations:** 1 The Newcastle upon Tyne Hospitals NHS Foundation Trust, Newcastle upon Tyne, United Kingdom; 2 Institute of Health and Society, Newcastle University, Newcastle upon Tyne, United Kingdom; 3 Health Services Research Unit, National Institute for Health and Welfare, Helsinki, Finland; 4 Health Services Research Unit, University of Aberdeen, Foresterhill, Aberdeen, United Kingdom; 5 Centre for Practice-Changing Research, Ottawa Hospital Research Institute, The Ottawa Hospital, Ottawa, Ontario, Canada; 6 College of Life Sciences and Medicine, University of Aberdeen, Foresterhill, Aberdeen, United Kingdom; Tehran University of Medical Sciences, Iran (Republic of Islamic)

## Abstract

**Background:**

Although most people with Type 2 diabetes receive their diabetes care in primary care, only a limited amount is known about the quality of diabetes care in this setting. We investigated the provision and receipt of diabetes care delivered in UK primary care.

**Methods:**

Postal surveys with all healthcare professionals and a random sample of 100 patients with Type 2 diabetes from 99 UK primary care practices.

**Results:**

326/361 (90.3%) doctors, 163/186 (87.6%) nurses and 3591 patients (41.8%) returned a questionnaire. Clinicians reported giving advice about lifestyle behaviours (e.g. 88% would routinely advise about calorie restriction; 99.6% about increasing exercise) more often than patients reported having received it (43% and 42%) and correlations between clinician and patient report were low. Patients’ reported levels of confidence about managing their diabetes were moderately high; a median (range) of 21% (3% to 39%) of patients reporting being not confident about various areas of diabetes self-management.

**Conclusions:**

Primary care practices have organisational structures in place and are, as judged by routine quality indicators, delivering high quality care. There remain evidence-practice gaps in the care provided and in the self confidence that patients have for key aspects of self management and further research is needed to address these issues. Future research should use robust designs and appropriately designed studies to investigate how best to improve this situation.

## Introduction

The current epidemic in Type 2 diabetes is largely being driven by an ageing population and by obesity [1]. The trend for more services to be delivered in primary care is UK government policy and is a cornerstone of modernising the NHS [2]. Most people with Type 2 diabetes no longer routinely attend hospital specialist clinics and receive their diabetes care from their primary care practice teams.

There have been a number of studies exploring the provision of primary care for patients with diabetes. These have largely focussed on the structure of care suggesting that whilst the organisational infrastructure for delivering care to patients with diabetes is in place [3], there is still variation in performance and room for improvement in the quality of care [4], [5]. Some of the variation in care has been shown to be associated with factors such as practice size and socioeconomic deprivation [6] but features such as dedicated clinic provision, staff numbers and training were not associated with compliance of process or outcome of care [4].

Policy support for diabetes care has been provided by the National Service Framework (NSF) [7], National Institute for Health and Clinical Excellence (NICE) guidelines [8] and the implementation of the Quality and Outcomes Framework (QOF; http://www.ic.nhs.uk/statistics-and-data-collections/audits-and-performance/the-quality-and-outcomes-framework accessed 2012 July 9th) which provides incentives for practice performance. Analysis of the QOF data suggests that whilst the care of patients with diabetes has improved, it is difficult to understand how much of this has been due to QOF [9]. Glycaemic control may have improved as a consequence of QOF but not in patients with type 2 diabetes and high HbA1c levels, and more stringent QOF thresholds might be needed in order to produce further improvement [10], [11].

All of these studies rely on either routinely available data or physician report. Several of the key behaviours required in diabetes care are not well recorded in routine clinical sources (such as primary care records) and their most reliable data source may be patients themselves. There are no comprehensive published data on the processes of care delivery for patients with diabetes cared for in primary care.

As one part of a larger study [12], [13] we have previously reported the organisational structure and intermediate outcomes of diabetes care across 99 UK primary care practices (74 in England, 13 in Scotland, four in Wales, and eight in Northern Ireland). The study was designed to better understand the quality of care patients with diabetes received through the performance of six key behaviours. These behaviours covered prescribing to control blood pressure and HbA1c (in patients with poor control), three advising behaviours (for weight management, self-management and general education) and one examining behaviour (foot examination). Practice attributes and a range of individually reported clinician measures were assessed at baseline; measures of clinical outcome were collected over the ensuing 12 months and a number of proxy measures of behaviour (including patient report) were collected at 12 months.

Our analysis of this data found that whilst QOF scores were generally high (with mean practice level percentage achievement rates of over 90% for 12 of the 15 clinical indicators), the mean percentage achievement rates for tight blood pressure control and tight HbA1c control were lower (80% and 68% respectively). Forty-nine practices had one or more clinicians trained to diploma level in diabetes care. Seventy-one practices had a dedicated diabetes clinic. Access to specialist support was variable. Most practices could access a diabetes nurse specialist (53 via secondary care, 28 via primary care) but GPs with a specialist interest in diabetes were rare (not available to 79 practices). Only 23 had access to a diabetes centre in secondary care and 44 practices reported having access to a specialist diabetologist. Forty-two practices did not have access to a dietician and 37 did not have access to a podiatrist.

Against this background of infrastructure and performance this paper presents further findings on the provision and receipt of care for patients with diabetes, as reported by healthcare professionals and patients. Specifically, we aimed to investigate the care of patients with Type 2 diabetes from the perspective of patients and health professionals, and to assess the extent to which the care that primary care clinicians report providing is associated with the care that people with Type 2 diabetes report receiving.

## Methods

### Setting and Subjects

Primary care practices were recruited from the UK Medical Research Council General Practice Research Framework (MRC GPRF). The UK MRC GPRF is a network of UK-based primary care practices interested in research that are broadly representative of UK primary care [13]. Participants were all the clinical members of the primary care team and patients registered with the practices recruited to the study.

### Patient Questionnaire

People with Type 2 diabetes were recruited by receiving and returning an anonymous questionnaire, which was derived from the UK NHS Healthcare Commission questionnaire used in the 2006 national survey of people with diabetes. It asked about the location of delivery of care patients had received, the content of that care and how confident they felt about managing their diabetes. A random sample of 100 adults with diabetes per practice was invited. If a practice had fewer than 100 patients with diabetes all were invited.

### Clinician Postal Questionnaire

All GPs and nurses in each practice were sent a questionnaire between September and December 2008. Questions were asked about: the provision of advice about weight management to patients with a BMI >30; providing self-management advice and providing general education. Clinicians were asked to prioritise their behaviour if pressed for time. Reminders were sent to non-responders at two and four weeks.

### Analysis

Descriptive statistics were used to calculate proportions and Pearson’s Correlation Coefficient to assess the relationship between patients’ and clinicians’ responses.

### Ethics Approval

Informed consent for clinicians was provided at the practice and individual level. Practices discussed participation in the study within the practice then returned a written consent form on behalf of the practice. Questionnaires with information sheets and written consent forms were then distributed to individual clinicians in consenting practices. For the patient questionnaire, patients were provided with information sheets and were informed of the anonymity of their responses to their practice and the study. To maintain anonymity, patients were informed that return of the questionnaire was taken as informed consent to participate. The ethics committee approved of the consent procedure for clinicians and patients. The study was approved by Newcastle and North Tyneside 2 Research Ethics Committee, REC reference number 07/H0907/102.

## Results

Eighty-six practices participated in the patient survey and the patient response rate was 41.8%. The clinician questionnaire was sent to 843 clinical staff in 99 practices. Completed questionnaires were returned by 326/361 (90.3%) primary care doctors and 163/186 (87.6%) nurses who indicated that they were involved in diabetes care.

### Patient Questionnaire

The mean age of respondents was 67.0 years with 11.9% reporting using insulin, 73.9% using tablets, 59.8% diet and 29.6% physical activity to help control their diabetes.

A practice mean (SD) of 93.9% (5.8) of Type 2 patients reported attending their primary care practice for their annual check. All but 1.8% (2.2) reported having attended for a diabetes check up in the last 12 months (where blood test results and treatment were reviewed); of those attending 29.1% (17.3) reported being seen once, 47.3% (15.1) twice and 18.7% (11.5) three or more times. The majority of patients (89.2% (7.0)) reported having had retinal photography and 85.4% (9.2) reported having had their bare feet examined. Only 18.7% (16) reported having seen a dietician. When asked if they had ever been offered an opportunity to attend an education or training course, 20.6% (13.3) reported having been offered this and 12.6% (9.2) reported participating in a course.

Table 1 and 2 show the practice mean (SD) percentages of patients reporting having received various elements of care. Almost two-thirds of patients reported receiving general and personalised advice. In order to identify patients reporting a normal BMI and who should thus be less likely to receive weight modification advice, responses were categorized by BMI. BMI was calculated from self-reported weight and height within the patient questionnaire. For a sub-set of 1006 patients from 41 practices we could use an anonymous linkage code to compare the BMI calculated from the self-reported data in the questionnaire with that recorded in their clinical records. The agreement between their self-reported data and that in the clinical record was good (intraclass correlation coefficient  = 0.79 (95% CI: 0.76, 0.82).) In the 63% of patients in whom BMI could be calculated from their self reported height and weight those patients with higher BMI consistently reported higher rates of receiving advice about diet but the range of responses remained wide. Over 40% of all patients had not agreed a plan to manage their diabetes, discussed their goals or received advice about levels of physical activity and the reported levels of receiving advice on aspects of diabetes care were under 50%.

**Table 1:** Practice mean (SD) percentage of patients reporting having received elements of care.

Thinking about the last 12 months, when you received care for yourdiabetes from a doctor or nurse	Mean (SD)% Yes*	BMI <25(n 454)	BMI 25–30(n 877)	BMI >30(n 924)
Were you provided with general information about diabetes?	68.3 (10.1)			
Were you given advice about how YOU should manage YOUR diabetes?	64.0 (11.0)			
Were you given advice about how to manage your weight?	47.8 (13.8)	28.5 (25.4)	44.8 (19.6)	61.1 (20.2)
Were you given advice about eating less to manage your weight?	43.0 (13.2)	19.5 (19.1)	39.8 (20.9)	57.2 (18.1)
Were you given written information (e.g. a leaflet) about managing your weight?	41.2 (13.5)	31.9 (22.9)	39.3 (19.0)	45.3 (19.0)
Were you given advice about doing more exercise to manage your weight?	42.1 (12.7)	21.0 (21.3)	39.6 (19.3)	54.4 (18.6)
Were you asked to see a dietician to discuss managing your weight?	24.4 (16.2)	16.0 (18.9)	20.0 (18.3)	31.3 (21.9)
Were you asked to see a dietician to discuss managing your blood sugar?	18.9 (11.7)			
Was it suggested to you to attend a gym to help manage your diabetes?	10.4 (8.7)			
Was it suggested to you to attend a weight loss organisation?	9.5 (8.5)	1.7 (5.7)	5.1 (8.2)	15.2 (14.3)
Were you offered or did you receive “exercise on prescription” to helpmanage your diabetes?	7.2 (7.2)			
Were you prescribed a drug to help you lose weight?	6.0 (5.5)	1.1 (4.7)	2.1 (4.5)	10.8 (11.4)
Did this information help you to better understand diabetes?	62.8 (10.7)			
		**Almost** **always**	**Some** **of the time**	**Rarely/not** **at all**
Did you agree when your next appointment would be?		54.0 (13.4)	14.5 (6.7)	22.7 (10.2)
Did you agree a plan to manage your diabetes over the next 12 months?		29.4 (12.4)	19.8 (7.8)	41.4 (12.3)
Were you given personal advice about the kinds of food to eat?		23.6 (9.1)	33.8 (11.1)	34.1 (11.1)
Did you discuss your ideas about the best way to manage your diabetes?		22.9 (8.6)	37.2 (8.9)	31.4 (12.1)
Did you discuss your goals in caring for your diabetes?		17.0 (7.8)	30.0 (10.9)	42.1 (11.3)
Were you given personal advice about your levels of physical activity?		15.9 (8.7)	32.2 (8.2)	41.7 (13.4)
Were you given the chance to discuss different medications?		15.4 (6.9)	25.1 (7.3)	49.1 (11.4)

* Between 1.1 and 4.0% responded “Don’t know” to each question.

**Table 2:** Practice Mean (SD) percentage of patients reporting having received advice about elements of care.

In the past 12 months, did you get advice about any of the following with a GP or nurse in relation to your diabetes?	Mean (sd) % Yes
Getting your eyes checked	76.0 (8.3)
Checking and looking after your feet	72.2 (10.9)
The impact of cholesterol levels on your diabetes	45.9 (12.8)
The reasons for taking prescribed medicines to manage your diabetes	44.1 (12.3)
The long term health effects of your diabetes	42.9 (11.5)
The impact of blood pressure levels on your diabetes	42.7 (11.5)
How drinking alcohol can affect your diabetes	40.7 (12.9)
Getting to and keeping to a certain weight	38.2 (12.3)
What to expect if your blood glucose drops too low	33.6 (11.7)
The effects of being ill, e.g. having flu, on managing your diabetes	32.6 (11.4)
The causes of diabetes	31.0 (10.2)
What to do to manage your symptoms	28.9 (11.5)
The effects of stress on your diabetes	24.9 (9.8)
The effects of tiredness on your diabetes	24.5 (9.8)

How did you get this advice?	
Verbally	67.1 (9.6)
GP/nurse gave you a leaflet or other printed material	37.0 (11.1)
The Diabetes UK website	5.3 (3.5)
Other:	2.4 (2.6)

When asked about their confidence in managing their diabetes (Table 3), patients responded on a five-point scale of 1 (Not at all confident) to 5 (very confident). Confidence was high for getting their eyes checked, looking after their feet and “managing their diabetes”. A minority reported having no confidence in their understanding about: what to do if blood glucose levels drop, impact of cholesterol levels, impact of blood pressure levels and the effects of stress on diabetes and overall a median (range) of 21% (3% to 39%) of patients reporting being not confident about various areas of diabetes self management.

**Table 3:** Practice mean (SD) values for patients’ reported levels of confidence that they can deal with elements of diabetes care.

How confident are you that youunderstand…	Not at allconfident				Very confident	Diabetes Duration#
…what to expect if your blood glucosedrops too low?	21.9 (8.2)	10.4 (11.0)	16.1 (5.7)	15.5 (7.3)	26.6 (7.6)	<0.0001
…the reasons for taking prescribed medicinesto manage your diabetes	7.4 (4.2)	6.3 (3.9)	14.6 (10.9)	18.1 (6.9)	39.1 (9.7)	<0.0001
…the long term health effects of your diabetes?	11.6 (5.8)	8.8 (4.6)	17.8 (10.7)	20.5 (7.9)	31.9 (8.8)	<0.0001
…the impact of cholesterol levels on yourdiabetes?	16.1 (7.3)	12.9 (5.5)	16.7 (6.2)	17.9 (6.9)	27.3 (11.0)	0.0003
…the impact of blood pressure levels on your diabetes	16.5 (7.3)	12.8 (6.5)	16.3 (6.5)	17.3 (6.8)	28.0 (11.0)	0.0002
…how drinking alcohol can affect your diabetes?	12.0 (5.6)	9.0 (4.8)	13.4 (5.7)	17.2 (6.6)	36.7 (10.7)	0.0004
…the effects of stress on your diabetes?	22.9 (7.8)	14.9 (10.5)	16.2 (6.8)	14.0 (5.6)	21.6 (7.2)	<0.0001
…the effects of tiredness on your diabetes?	24.5 (8.1)	14.4 (5.4)	17.5 (11.0)	12.6 (5.5)	21.6 (7.3)	0.0003

How confident are you that you can …						
…manage your diabetes?	3.8 (3.2)	5.8 (4.7)	19.3 (7.1)	26.6 (10.5)	40.6 (10.2)	<0.0001
…get to and keep to a certain weight?	14.1 (6.1)	12.5 (6.3)	23.3 (7.8)	20.5 (10.8)	24.1 (8.1)	0.6580
…get your eyes checked?	1.4 (2.3)	1.8 (2.0)	5.1 (4.1)	13.5 (6.3)	75.6 (8.6)	0.0324
…check and look after your feet?	3.1 (3.3)	3.6 (3.4)	9.1 (5.5)	16.3 (6.3)	64.8 (11.1)	0.0008
…manage your exercise/activity levels?	10.2 (5.5)	10.4 (6.2)	22.0 (7.7)	21.0 (11.1)	30.4 (7.5)	0.6188
…manage your diet?	5.5 (4.0)	8.9 (4.4)	23.1 (8.2)	24.5 (11.5)	34.0 (8.3)	0.2422
…take your medication as prescribed?	1.77 (2.05)	0.95 (1.73)	3.95 (3.09)	10.56 (5.58)	73.26 (7.18)	0.0001

# comparison of those scoring “confident” or “very confident” for patients who have had diabetes less than two years versus those who have had diabetes more than two years.

When comparing the impact of duration of diabetes (≤2 yrs versus >2 yrs) on the proportion of patients scoring “confident” or “very confident”, patients who had diabetes for >2 years reported significantly higher ratings for all of the “confidence in knowing” questions but for only four of the seven “confident that you can” questions. For “confident that you can” questions on weight management, diet and exercise there was no effect of duration.

### Healthcare Professional Reported Behaviour

Clinicians were asked what specific behaviours they routinely included as part of three more generally labelled advising behaviours (Tables 4, 5, 6). When providing advice about weight management to patients who’s BMI is above target, nearly all healthcare professionals would include increasing exercise and calorie restriction. Across all eight weight management behaviours 63% of respondents reported routinely including advice on at least five. When providing advice on the self-management of diabetes respondents most commonly reported routinely individualising advice, or advised referral to a dietician. Across the six patient self-management behaviours 72% of respondents reported routinely including advice on at least four areas and across nine general education behaviours 69% of respondents endorsed seven or more areas of advice as routinely offered.

**Table 4:** Percentage of clinicians responding to components of their routine advice about weight management, self management and general education.

Questions in the clinician questionnaire	% Yes(N 487to 491)	Which ONEwould youdo?## (N 416)	Corresponding questions inpatient questionnaire	% “Yes” or“Almost Always”	Correlation between patient and clinician responses#
*“Providing advice about weight management to patients whose BMI is above target is something for me that routinely includes …”*			*“Thinking about the last 12 months,* *when you received care for your diabetes* *from a doctor or nurse…”*		
Advising about increasing exercise	99.6	33.4	Were you given advice about doingmore exercise to manage your weight?	54.4	−0.20
Advising about calorie restriction	85.7	15.9	Were you given advice about eatingless to manage your weight?	57.1	−0.21**
Providing a printed leaflet	64.2	16.1	Were you given written information(e.g. a leaflet) about managing yourweight	45.3	0.03
Referral to a dietician	62.3	8.4	Were you asked to see a dietician todiscuss managing your weight?	31.3	0.27**
Prescribing exercise	56.7	2.2	–		
Referral to the practice nurse	48.2	17.3	–		
Suggesting a commercial weight loss organisation	45.4	1.9	Was it suggested to you to attend aweight loss organisation?	15.2	0.05
Suggesting a commercial gym/exercise organisation	39.4	0.2	–		
Other	27.4	4.6	–		

# Correlation is with proportion of people responding and BMI>30.

## Question stem “If pressed for time which ONE would you do?”; figure is % endorsing that response.

** p<0.01.

**Table 5:** Percentage of clinicians responding to components of their routine advice about self management.

Questions in the clinicianquestionnaire	% Yes (N 487 to 491)	Which ONEwould youdo?## (N 416)	Corresponding questions inpatient questionnaire	% “Yes” or “Almost Always”	Correlation between patient and clinician responses
*Providing patients with advice on the self-* *management of their diabetes is something* *that for me routinely includes …*			*Again thinking about the last 12 months, when you received care for your diabetes from a doctor or nurse …’*		
Giving advice that takes account ofindividual circumstances	86.2	22.7	Did you agree a plan to manage yourdiabetes over the next 12 months?	29.4%	0.14
Advising about the nutritional contentof their diet	84.9	18.5	Were you given personal adviceabout the kinds of food to eat?	23.6%	0.10
Referral to a dietician	73.5	5.3	In the last 12 months have youseen a dietician?	18.7%	0.37**
Referral to the practice nurse	62.7	40.4			
Providing disposable equipmentfor self-monitoring of blood glucose	61.0	1.85			
Suggesting NHS course for trainingpatients with diabetes in selfmanagement	50.1	9.5			
Other	15.4	1.85			

## Question stem “If pressed for time which ONE would you do?”; figure is % endorsing that response.

** p<0.01.

**Table 6:** Percentage of clinicians responding to components of their routine advice about general education.

Questions in the clinicianquestionnaire	% Yes(N 487 to 491)	Which ONEwould youdo?## (N 416)	Corresponding questions inpatient questionnaire	% “Yes” or “Almost Always”	Correlation between patient and clinician responses
*“Providing patients with general* *education about diabetes is something that* *for me routinely includes … “*			*“Thinking about the last 12 months,* *when you received care for your diabetes* *from a doctor or nurse…”*		
How the patient is involved incontrolling diabetes	96.9	28.6	How you should manage your diabetes	64.0	−0.04
			Best way to manage your diabetes	22.9	0.06
			Agree a plan to manage your diabetesover the next 12 months	29.4	0.09
			What to do to manage your symptoms	28.9	−0.01
Ensuring that they understand	91.3	19.5	Information help you better understanddiabetes	62.8	−0.05
What the symptoms of diabetes are	92.3	2.3	Provided with general informationabout diabetes	68.3	0.04
Medical management	89.2	11.7	Given the chance to discuss differentmedications	15.4	0.18
			Reasons for taking prescribed medicinesto manage your diabetes	44.1	0.23
Providing a leaflet/printed materials	85.1	21.0	Gave you a leaflet or other printermaterial	37.0	0.08
The cause of diabetes	78.7	1.6	The causes of diabetes	31.0	0.12
The time course of diabetes	66.8	0.3	Long term health effects of yourdiabetes	42.9	0.03
Recommending Diabetes UK	66.8	6.0	Ever visited Diabetes UK website	19.1	0.14
Recommending a diabetes educationcourse	52.9	5.7	Offered opportunity to attendeducation/training course	20.6	0.42**
Other	12.7	3.1			

## Question stem “If pressed for time which ONE would you do?”; figure is % endorsing that response.

** p<0.01.

### Comparison of Clinician and Patient Responses

When clinician responses were compared with the responses to corresponding questions from the patient questionnaire (Tables 4, 5, 6) patient responses were invariably lower and the correlations between the two were low. Though the rates were different the correlation was statistically significant for four questions, which included the questions about weight management, referral to a dietician, (asked in the context of both weight management and self management) and attendance at a patient education course.

## Discussion

This study presents a unique overview of the state of provision of diabetes care in primary care practices in the UK. In the face of apparently high levels of achievement in QOF we document considerable variation in the delivery and receipt of care from the perspectives of the health care professionals and patients. This is set against the backdrop of a recent English report confirming widespread variation but demonstrating poor levels of performance [14]. The report documents that whilst performance on individual quality indicators was comparable with figures from this study, the proportion of patients receiving more than six of the nine elements of diabetes care suggested in national standards was under 90% and the proportion receiving all nine was under 50%. They also report sub-optimal control of risk factors, though by the nature of their data, they can make no allowance for clinician actions in response to raised risk factor values. The five areas of care that we included in this study are recognised as important elements of care but three of them do not feature in the routine quality indicators. Given that there is some lack of provision (and reported receipt) across these three then it is likely that they represent further areas where care should be improved.

We present the range of dimensions of care received and the (lack of) correspondence between what clinicians report delivering and patients report receiving. This mismatch between patient and healthcare professionals’ perceptions is likely to be a real phenomenon and highlights important areas where diabetes care can be improved. Most people with diabetes reported receiving their diabetes care mainly in primary care, they reported high rates of having regular physical checks of their eyes and feet but reported lower rates of receiving advice on weight management, self-management and education. A large minority lacked confidence in their ability to manage some aspects of their diabetes; healthcare professionals reported consistently high rates of discussing these areas. The quality of provider communication and involving patients in decision-making has previously been shown to predict patients’ reported diabetes self-management capability, suggesting possible opportunities for improving care and self-management [15].

The difference in rates of reporting between patients and health care professionals could be due to recall bias (with patients forgetting advising behaviours that might be less memorable than an eye check). This is supported by the fact that, for some of the areas of care those patients who had had diabetes for longer reported greater recall. However, given that many of the areas described are of continuing importance this highlights the need to consider what patients need to know and how best to deliver this as well as a role for on-going checks of what patients understand.

From the perspective of the healthcare professionals their higher rates of self-reported performance could represent a desirability response bias (recognising desirable behaviours and subconsciously over-reporting). It may be that clinicians may be tailoring their advice for experienced patients yet reporting their behaviour in relation to their overall population of patients. They may deliver advice to those patients they perceive need it and not to those they know have received the advice before. Even so, patient confidence rates for enacting key behaviours were not influenced by duration of diabetes and were positive for just over half of respondents. The Audit report [14] certainly reports variability but cannot reflect the complexity of patient management and may, from the perspective of improving care in primary care practices, not be reflecting the complexity of patient management. Of the three care processes most often missed (retinal screening, foot examination, urinary ACR) one is not under the control of the practices (retinal screening), one is sometimes performed by non-practice staff (foot examination) and one is reliant on patients remembering to bring a urine sample (urinary ACR). In terms of level of control of risk factors the audit data could take no account of current management or management actions within the practices. Therefore, improving care will be a complex and multi-faceted undertaking.

The limitations of this study include: practices were recruited from a research network and so might be atypical in terms of the care they offer and may offer better care than that delivered in practices that are not in research networks. This would suggest that even higher proportions of patients may be receiving care of a lower standard than that reported here. The response rate to the patient survey was below 50%; patients who responded to the patient survey were anonymous and we have no means of analysing whether or not they were typical of the rest of the patients in the practices from which they come. Their responses have to be regarded with caution in the light of this possible response bias.

### Conclusions

Primary care practices have organisational structures in place and are, as judged by practice level routine quality indicators, delivering high quality care. However, at an individual patient level care may not be as good. Reported rates of performing key management behaviours differ between clinicians and patients with clinicians reporting higher rates of performing than patients report receiving; patients report low levels of confidence for key self-management behaviours. Future research should use robust designs and appropriately designed studies (documenting outcomes at a patient as well as practice level) to investigate how best to improve this situation.
