# Correction: Diabetes Care Provision in UK Primary Care Practices

**DOI:** 10.1371/annotation/1957ad3b-e192-4faa-bf4c-5dce22c5560e

**Published:** 2012-08-03

**Authors:** Gillian Hawthorne, Susan Hrisos, Elaine Stamp, Marko Elovainio, Jill J. Francis, Jeremy M. Grimshaw, Margaret Hunter, Marie Johnston, Justin Presseau, Nick Steen, Martin P. Eccles

Please note that cited reference [10] is a duplicate of cited reference [9]. Both cite the same study by Calvert et al. (BMJ 2009;338:b1870) 

